# Extracellular Vesicles from Human iPSC‐derived NSCs Protect Human Neurons Against Aβ‐42 Induced Neurodegeneration, Mitochondrial Dysfunction and Tau Phosphorylation

**DOI:** 10.1002/alz.093150

**Published:** 2025-01-03

**Authors:** Shama Rao, Leelavathi N Madhu, Roshni S Babu, Advaidhaa Nagarajan, Esha Narvekar, Raghavendra Upadhya, Ashok K Shetty

**Affiliations:** ^1^ Institute for Regenerative Medicine, Department of Cell Biology and Genetics, School of Medicine, Texas A&M University Health Science Center, College Station, Texas, USA., College Station, TX USA; ^2^ Institute for Regenerative Medicine, Department of Cell Biology and Genetics, School of Medicine, Texas A&M University Health Science Center, College Station, TX USA

## Abstract

**Background:**

Alzheimer’s disease (AD) is characterized by the accumulation of amyloid‐beta (Aβ) in the extracellular space, which leads to various adverse effects such as oxidative stress, neuroinflammation, mitochondrial dysfunction, tau phosphorylation, synapse loss, and neurodegeneration. Therefore, therapeutic interventions that can reduce Aβ‐toxicity and slow down the progression of cognitive dysfunction in AD have significance. One promising approach is to use extracellular vesicles (EVs) that are released by neural stem cells (NSCs) derived from human induced pluripotent stem cells (hiPSCs). These EVs contain therapeutic proteins and miRNAs that can help protect against Aβ‐induced pathological changes in the brain, providing a potential avenue for neuroprotection.

**Method:**

This study first isolated and characterized EVs secreted from hiPSC‐NSCs by chromatographic methods. Next, mature human neurons generated from 2 different hiPSC lines were exposed to 1‐µM Aβ42 oligomers with different concentrations of hNSC‐EVs. The protective effects of EVs on neurons exposed to Aβ were quantified using MTT and live/dead cell assays and counting of surviving MAP‐2+ neurons. The extent of oxidative stress was investigated via measurements of malondialdehyde (MDA) and protein carbonyls (PCs) and the expression of genes encoding iNos and Cox2. Furthermore, the expression of genes and/or proteins linked to pro‐apoptotic (Bad, Bax) and antiapoptotic (Bcl2) effects, mitochondrial respiratory chain (Nudfs6, Nudfs7, Sdha, Sdhb, Cyc1, Bcs11, Cox6a1, Atp6ap1), autophagy (Beclin 1 and MAPILC3B) were quantified. Total tau and p‐tau were also measured.

**Result:**

The addition of hiPSC‐NSC‐EVs to mature human neuronal cultures exposed to Aβ reduced the extent of Aβ‐induced neurodegeneration. When human neurons were exposed to Aβ‐42 alone, it increased pro‐apoptotic genes (Bad, Bax) and reduced the expression of an antiapoptotic gene (BCL‐2). However, adding hNSC‐EVs to Aβ‐42 exposed neurons normalized the expression of pro‐ and antiapoptotic genes to control levels, reduced oxidative stress markers, prevented Aβ‐42‐induced hyperactivation of mitochondria and decreased autophagy, reduced the phosphorylation of tau.

**Conclusion:**

Treating human neurons with hiPSC‐NSC‐EVs can significantly decrease the various pathological changes caused by Aβ exposure. Supported by a grant from the National Institute for Aging (1RF1AG074256‐01A1 to A.K.S.)

